# What Is the Efficacy of Sotrovimab in Reducing Disease Progression and Death in People with COVID-19 during the Omicron Era? Answers from a Real-Life Study

**DOI:** 10.3390/v15081757

**Published:** 2023-08-17

**Authors:** Andrea De Vito, Agnese Colpani, Mariacristina Poliseno, Lucia Diella, Francesco Rosario Paolo Ieva, Alessandra Belati, Roberto Papale, Sergio Babudieri, Laura De Santis, Annalisa Saracino, Sergio Lo Caputo, Giordano Madeddu

**Affiliations:** 1 Unit of Infectious Diseases, Department of Medicine, Surgery, and Pharmacy, University of Sassari, 07100 Sassari, Italy; colpani.agnese@gmail.com (A.C.); giordano@uniss.it (G.M.); 2 S.C. Malattie Infettive, Dipartimento di Medicina Clinica e Sperimentale, University of Foggia, 71100 Foggia, Italy; polisenomc@gmail.com (M.P.); sergio.locaputo@unifg.it (S.L.C.); 3 Clinic of Infectious Diseases, Department of Precision and Regenerative Medicine and Ionian Area—(DiMePRe-J), University of Bari “Aldo Moro”, Piazza Giulio Cesare n. 11, 70100 Bari, Italya.belati@studenti.uniba.it (A.B.); l.desantis29@studenti.uniba.it (L.D.S.); annalisa.saracino@uniba.it (A.S.)

**Keywords:** sotrovimab, SARS-CoV-2, COVID-19, mortality, disease progression

## Abstract

(1) Introduction: Since May 2021, sotrovimab has been available in Italy for early treatment of SARS-CoV-2 infection and to prevent disease progression. However, some in vitro studies have questioned its efficacy on Omicron variants. Therefore, we aim to further investigate the efficacy of sotrovimab in real-life settings. (2) Methods: We conducted a retrospective study collecting medical records of people with SARS-CoV-2 infection evaluated in the infectious diseases units of Sassari, Foggia, and Bari, Italy. We included people with SARS-CoV-2 infection treated with sotrovimab and people who did not receive any treatment in 2022. The primary study outcome was to evaluate the efficacy of sotrovimab in reducing disease progression (defined as the necessity of starting oxygen supplementation) and COVID-19-related death. The secondary outcome was to evaluate the safety of sotrovimab. (3) Results: We included 689 people; of them, 341 were treated with sotrovimab, while 348 did not receive any treatment. Overall, we registered 161 (23.4%) disease progressions and 65 (9.4%) deaths, with a significant difference between treated and not-treated people (*p* < 0.001). In the multivariate logistic regression, increasing age [OR for ten years increasing age 1.23 (95%CI 1.04–1.45)] was associated with a higher risk of disease progression. In addition, cardiovascular disease [OR 1.69 (1.01–2.80), fever [OR 3.88 (95%CI 2.35–6.38)], and dyspnea [OR 7.24 (95%CI 4.17–12.58)] were associated with an increased risk of disease progression. In contrast, vaccination [OR 0.21 (95%CI 0.12–0.37)] and sotrovimab administration [OR 0.05 (95%CI 0.02–0.11)] were associated with a lower risk of developing severe COVID-19. Regarding mortality, people with older age [OR for ten years increasing age 1.36 (95%CI 1.09–1.69)] had a higher risk of death. In addition, in the multivariate analysis, cardiovascular disease lost statistical significance, while people on chemotherapy for haematological cancer [OR 4.07 (95%CI 1.45–11.4)] and those with dyspnea at diagnosis [OR 3.63 (95%CI 2.02–6.50)] had an increased risk of death. In contrast, vaccination [OR 0.37 (95%CI 0.20–0.68)] and sotrovimab treatment [OR 0.16 (95%CI 0.06–0.42)] were associated with lower risk. Only two adverse events were reported; one person complained of diarrhoea a few hours after sotrovimab administration, and one had an allergic reaction with cutaneous rash and itching. (4) Conclusions: Our study showed that sotrovimab treatment was associated with a reduction of the risk of disease progression and death in SARS-CoV-2-infected people, 70% of whom were over 65 years and a with high vaccination rate, with excellent safety. Therefore, our results reinforce the evidence about the efficacy and safety of sotrovimab during the Omicron era in a real-world setting.

## 1. Introduction

Severe Acute Respiratory Syndrome Coronavirus-2 (SARS-CoV-2) is a novel coronavirus, first identified in Wuhan, China in late 2019 [1]. This virus belongs to the broader family of coronaviruses, including SARS-CoV and MERS-CoV, both of which have caused severe respiratory diseases in humans [2,3]. SARS-CoV-2, however, has proved to be significantly more infectious, resulting in a widespread global pandemic [4,5].

Coronavirus Disease 2019 (COVID-19), caused by SARS-CoV-2, initially presents with a broad spectrum of symptoms, ranging from mild to severe [6]. Common symptoms include fever, cough, and shortness of breath, but a wide variety of other symptoms, such as loss of taste and smell, fatigue, skin lesions, and gastrointestinal symptoms, can also occur [7,8,9,10,11]. In some cases, the disease progresses to severe pneumonia, acute respiratory distress syndrome (ARDS), and multi-organ dysfunction, leading to significant morbidity and mortality [12,13,14].

As the SARS-CoV-2 virus has spread worldwide, it has continued to mutate, as is common with RNA viruses, leading to the development of several variants of concern (VOCs) [15]. These VOCs, such as Alpha, Beta, Delta, and Omicron, have often demonstrated increased transmissibility, altered disease severity, and potential for immune escape [16]. In this regard, the Omicron variant has many mutations on the spike protein, the target for most vaccines and many therapeutic antibodies, raising concerns about vaccine and therapeutic efficacy [17,18,19,20].

In response to the global COVID-19 pandemic, an unprecedented global effort has developed several vaccines and therapeutics. Among antiviral therapies, at the end of 2021, monlupiravir, nirmatrelvir/ritonavir, and remdesivir were approved to reduce the risk of severe disease and death [21,22,23]. Monoclonal antibodies (mAbs) were designed to target the SARS-CoV-2 spike protein and prevent the virus from entering host cells. Sotrovimab is one such mAb, which has shown promising results in clinical trials, with the potential to reduce the risk of hospitalization and death in COVID-19 patients [24,25].

Sotrovimab works by binding to the SARS-CoV-2 spike protein, preventing the virus from entering host cells and thus inhibiting replication. Preliminary data have indicated its potential effectiveness against earlier VOCs. Still, with the arrival of the highly mutated Omicron variant, it is crucial to evaluate the real-world efficacy of sotrovimab in preventing severe disease and death [20,26]. Several in vitro studies have questioned the efficacy of sotrovimab against Omicron subvariants [27,28]. In this study, we aim to investigate the effectiveness of sotrovimab in a real-world context during the Omicron era.

## 2. Materials and Methods

We conducted a retrospective study collecting medical records of people with SARS-CoV-2 infection evaluated at the infectious diseases units of Sassari, Foggia, and Bari, Italy, between 1 January 2022, and 31 December 2022. We included people with SARS-CoV-2 infection treated with sotrovimab in 2022 and people who did not receive any treatment.

Inclusion criteria were (i) age 18 years or more; (ii) diagnosis of SARS-CoV-2 infection by polymerase chain reaction (PCR) or third-generation antigenic tests.

Exclusion criteria were the presence of a respiratory failure at the first evaluation (PaO_2_/FiO_2_ > 300).

We collected information on medical history, vaccination status, symptoms at admission, and therapies.

### 2.1. Treatment Prescription

In all three hospitals, people with SARS-CoV-2 infection were evaluated by an infectious diseases specialist, and those eligible for treatment received the prescription.

Prescriptions for antiviral therapies (such as molnupiravir, nirmatrelvir/ritonavir, and remdesivir) as well as monoclonal antibodies (including casirivimab/imdevimab and sotrovimab) followed the guidelines set forth by the Italian Drug Agency. The qualifications for administering these treatments included the recent emergence of symptoms (no more than 5 days for molnupiravir and nirmatrelvir/ritonavir, and no more than 7 days for remdesivir and monoclonal antibodies), absence of a requirement for supplementary oxygen, and a heightened likelihood of illness progression due to the existence of one or more chronic conditions such as: (i) obesity with a body mass index exceeding 30; (ii) diabetes mellitus with associated organ damage or hemoglobin A1c above 7.5%; (iii) renal failure; (iv) acute pulmonary disease; (v) significant heart disease; (vi) immune system disorders; (vii) malignancies. Exclusions encompassed: (i) an estimated glomerular filtration rate (eGFR) of under 30 mL/min/1.73 m^2^ (specifically for nirmatrelvir/ritonavir and remdesivir); (ii) gestation; (iii) progressed chronic hepatic disease. Furthermore, males prescribed molnupiravir had to commit to utilizing condoms for a minimum of three months if partnered with a fertile female, and fertile females had to agree to use condoms for a minimum of four days following the conclusion of treatment.

Regarding treatment, the decision rested with the treating physician if multiple options were appropriate. Factors influencing this decision included potential drug interactions, acute kidney dysfunction, a need for hospitalization, the capacity to ingest orally, and the fragility of the patient.

As for sotrovimab, eligible individuals to whom the drug was prescribed received a 500 mg dose of the medication in a single intravenous administration.

The rationale for withholding treatment included: (i) an absence of symptoms at the time of examination; (ii) the passage of more than seven days since symptoms first appeared; (iii) the personal preference of the patient.

### 2.2. Outcomes

The efficacy of sotrovimab to decrease the risk of disease progression (i.e., administration of oxygen, non-invasive ventilation, and death) was evaluated as the primary study endpoint. Furthermore, predictors of disease progression were investigated.

The secondary objective of our study was to evaluate the safety of sotrovimab and describe the adverse events registered.

### 2.3. Statistical Analysis

The distribution of quantitative data in our study was examined utilizing normality tests such as the Shapiro–Wilk test. Quantitative variables were provided for data representation regarding medians and the interquartile range (IQR), denoting the 25th and 75th percentiles, or mean and standard deviation (SD), according to the normality of distribution. Qualitative variables were represented by both absolute numbers and relative frequencies (percentages).

Differences between subgroups for quantitative data were identified using the Mann–Whitney U test. To evaluate differences in qualitative variables, we employed Pearson’s chi-square test or Fisher’s exact test, as appropriate. We sought to identify factors correlating with disease progression through logistic regression analysis.

The Kaplan–Meier curve and log-rank test were performed to describe hospital discharge after 7 and 14 days, stratifying by sotrovimab prescription.

Statistical significance was determined by a *p* value of less than 0.05 (two-tailed). The statistical software package STATA (version 16.1, StataCorp, College Station, TX, USA) was used to perform all the statistical analyses.

## 3. Results

We included 689 people; of them, 341 were treated with sotrovimab, while 348 had not received any treatment. People who received sotrovimab were significantly younger than people who had not received any treatment (median age 71.1 vs. 74.9 years, *p* = 0.0012), while there was no difference in the number of people aged 65 years or more (67.7% vs. 71%, *p* = 0.190).

Regarding comorbidities, in the sotrovimab group, there was a higher percentage of people with immunodepression, oncological disease, haematological cancer, transplant recipients, and those who had had a previous cerebrovascular accident. In contrast, in the not-treated group, there was a higher percentage of people with cardiovascular disease.

Regarding vaccination, we saw a statistically significant difference between the two groups, with a lower percentage of fully vaccinated people (at least two doses) in the not-treated group (78.4% vs. 85.3%, *p =* 0.019).

Looking at concomitant antiviral treatment, in the sotrovimab group, 61 (17.9%) people received monlupiravir, 9 (2.6%) received nirmatrelvir/ritonavir, and 26 (7.6%) received a 3-day course of remdesivir.

Overall, we registered disease progression in 23.4% of our patients, with a significant difference between the two groups [17 (5.0%) in sotrovimab group vs. 144 (41.4%) in the not-treated group, *p* < 0.001].

Furthermore, regarding death due to COVID-19, we registered lower mortality in people treated with sotrovimab (2.6% vs. 16.1, *p* < 0.001). The characteristics of our cohort are summarized in Table 1.

In the multivariate logistic regression, increasing age [OR for ten years increased age 1.23 (95%CI 1.04–1.45)] was associated with a higher risk of disease progression. In addition, cardiovascular disease [OR 1.69 (1.01–2.80)], fever [OR 3.88 (95%CI 2.35–6.38)], and dyspnea [OR 7.24 (95%CI 4.17–12.58)] were associated with an increased risk of disease progression. On the contrary, vaccination [OR 0.21 (95%CI 0.12–0.37)] and sotrovimab administration [OR 0.05 (95%CI 0.02–0.11)] were associated with a lower risk of having severe disease (Table 2).

Regarding mortality due to COVID-19, people with older age [OR for ten years increased age 1.36 (95%CI 1.09–1.69)] had a higher risk of death. In addition, in the multivariate analysis, cardiovascular disease lost statistical significance. At the same time, people on chemotherapy for haematological cancer [OR 4.07 (95%CI 1.45–11.4)] and those with dyspnea [OR 3.63 (95%CI 2.02–6.50)] had an increased risk of death. In contrast, vaccination [OR 0.37 (95%CI0.20–0.68)] and sotrovimab treatment [OR 0.16 (95%CI 0.06–0.42)] were associated with lower risk (Table 3).

**Figure 1:**
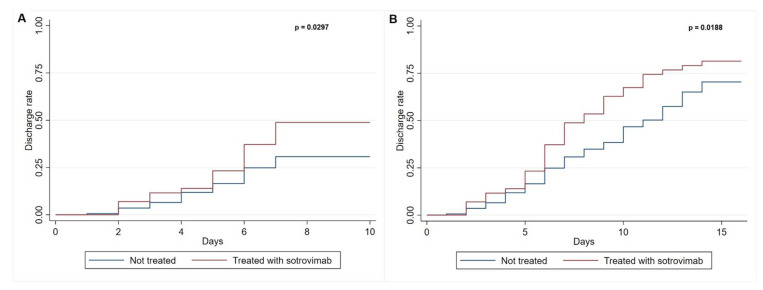
(**A**) 7-day hospital discharge of 215 people with SARS-CoV-2 infection. (**B**) 14-day hospital discharge of 215 people with SARS-CoV-2 infection.

A subanalysis of the impact of sotrovimab on the length of hospital stay was conducted. Among the 476 people admitted to hospital, we excluded 207 patients who had been infected during hospitalization for other causes; additionally, we excluded 43 people who died during hospitalization and 11 people who had been moved to another ward, as we were not able to collect their discharge data. Overall, we included 215 people. People treated with sotrovimab had a lower median hospital stay compared with those not treated [7.5 (IQR 5.5–11.5) vs. 11 (6–17), *p* value 0.0298]. In addition, people treated with sotrovimab had a higher discharge rate on days 7 and 14 than those without early treatment (Figure 1A,B).

Finally, regarding adverse events, only two were reported; one person complained of diarrhoea a few hours after sotrovimab administration, and one had an allergic reaction with cutaneous rash and itching.

## 4. Discussion

It is widely recognized that the Omicron variant of SARS-CoV-2 is more contagious and has the ability to evade immunity in individuals who have been previously vaccinated. However, the Omicron variant generally poses a lower risk of severe outcomes than the Delta variant. This is primarily due to certain intrinsic characteristics of the Omicron variant that make the infection less severe, as well as the protective effects of vaccination [29,30,31].

Despite the apparent decrease in disease severity associated with the Omicron variant, the high transmissibility of the virus has resulted in a sustained strain on healthcare systems. This is evident in the latest report from the Centers for Disease Control (CDC) in the United States, which indicates that the volume of emergency department visits, the average daily number of hospitalizations, and the rate of COVID-19-related deaths remain substantially unchanged [32]. Similar trends are being observed in Italy, where the number of new SARS-CoV-2 infections is approximately 30,000 per week, and the numbers of hospitalizations and deaths due to COVID-19 are still unacceptably high [33]. Regarding the prevalence of the Omicron variant in Italy, at the start of enrollment this was around 85%, reaching 96% on the 17 January 2022 [34].

Furthermore, despite the lower pathogenicity observed with the Omicron variant, recent Italian experiences have highlighted that in times of Omicron predominance, elderly individuals and those with underlying health conditions are still susceptible to hospitalization and poor outcomes, particularly when respiratory insufficiency is involved [35].

To address this ongoing challenge, it is essential to provide effective treatments that could prevent disease progression and death in such vulnerable populations.

Our retrospective study of medical records from people with SARS-CoV-2 infection evaluated at the infectious diseases units of Sassari, Foggia, and Bari provides several key findings. The study aimed to evaluate the effectiveness and safety of sotrovimab, an antiviral treatment for COVID-19, in reducing the risk of disease progression and mortality.

In general, our findings highlight the potential benefits of sotrovimab treatment for patients with SARS-CoV-2 infection, as anticipated by in vitro studies exploring the efficacy of sotrovimab against the Omicron variant [36]. We observed a significantly lower rate of disease progression and mortality among patients who received sotrovimab compared with those who did not receive any treatment. This is in line with previous studies which reported the potential effectiveness of sotrovimab in the management of SARS-CoV-2 infection. In a randomized clinical trial, the percentages of severe disease and death were much lower compared with our study, in both treated and not-treated patients. However, the unselected population of our cohort was significantly older (median age 72.4 years vs. 53.0), with a higher comorbidity burden [37].

A retrospective observational study by Piccicacco et al. conducted among outpatients showed a protective role of sotrovimab considering hospitalization and emergency department access [38]. A similar effect was demonstrated for remdesivir. Furthermore, in a large observational study, Cheng et al. compared a cohort not receiving monoclonal antibodies (*n* = 1,514,868) and a sotrovimab-treated cohort (n = 15,633) [39]. Although sotrovimab-treated individuals were older and with a higher proportion of high-risk conditions, in the cohort without monoclonal antibodies, 84,307 (5.57%) patients were hospitalized and 8167 (0.54%) deaths were identified, while in the sotrovimab cohort 418 (2.67%) patients were hospitalized and 13 (0.08%) deaths were identified. After adjusting for potential confounders, the sotrovimab cohort had a 55% lower risk of 30-day hospitalization or mortality (RR 0.45, 95%CI 0.41–0.49) and an 85% lower risk of 30-day mortality (RR 0.15, 95%CI 0.08–0.29).

Regarding high-risk populations, Chavarot et al. published their data on a particular subset of patients consisting of kidney transplant recipients, supporting the efficacy of sotrovimab in fragile patients with the Omicron variant [40]. A similar outcome was reported by Radcliffe et al. and Pinchera et al., again among solid organ (mainly kidney) transplant recipients [41,42]. In addition, Evans et al. investigated the efficacy of sotrovimab, molnupiravir, and nirmatrelvir/ritonavir among high-risk patients from the SAIL databank (more than 40% with immune deficiency). The adjusted HRs for hospitalization or death for patients treated with molnupiravir, nirmatrelvir/ritonavir, and sotrovimab were 0.49 (95%CI: 0.29–0.83), 0.59 (95%CI: 0.36–0.97), and 0.73 (95%CI: 0.55–0.98), respectively, with no indication of the superiority of one treatment over another. Interestingly, the reduction in hospitalizations and deaths found in the study were consistent with published pre-Omicron randomized controlled trials for sotrovimab, molnupiravir, and nirmatrelvir/ritonavir, despite being carried out when Omicron was the predominant variant in Wales [43].

In contrast, Woo et al. conducted a propensity-score-matched retrospective cohort study on 1254 people with COVID-19. Of them, 185 were treated with sotrovimab. The authors found no difference in the in-hospital mortality [44]. These data could be explained by the fact the majority of people had been treated in the intensive care unit (103/185, 55.7%), reinforcing the message that people with SARS-CoV-2 infection at risk of severe diseases must be treated as soon as possible.

Regarding the length of hospitalization, the sotrovimab-treated group showed a shorter hospital stay than those who had not received any treatment, and the estimated rate of hospital discharge at days 7 and 14 was higher in the sotrovimab-treated group. Similar results were reported by Aggarwal et al. [45]. In their study, people treated with sotrovimab had a halved mean length of hospital stay compared with people who were not treated.

Nevertheless, the retrospective nature of our study introduces potential biases that could influence the results. In addition, the choice of treatment was at the discretion of the attending physician, which might have resulted in selection bias. Therefore, we could not evaluate differences regarding disease progression between patients treated with sotrovimab alone or in combination with other antiviral compounds. Future analyses on larger databases are needed to evaluate the outcomes for people treated with sotrovimab and other antiviral treatments [46].

Our study provides valuable insights into the potential benefits of sotrovimab in reducing disease progression and mortality in patients with SARS-CoV-2 infection in the Omicron era, highlighting the importance of early therapeutic intervention and vaccination. These findings can contribute to current strategies for managing COVID-19 and prompt further research on the role of antiviral treatments in different patient populations.

## 5. Conclusions

Our study showed that sotrovimab treatment was associated with a reduction of the risk of disease progression and death in SARS-CoV-2-infected people 70% of whom were over 65 years and with a high vaccination rate, with excellent safety. Therefore, our results reinforce the evidence about the efficacy and safety of sotrovimab during the Omicron era in a real-world setting.

## Figures and Tables

**Table 1:** Characteristics of 689 people with SARS-CoV-2 divided by those treated with sotrovimab or not.

	Overall (689)	Sotrovimab (341)	Not Treated (348)	*p* Value
Age (years), median (IQR)	72.4 (60.9–80.0)	71.1 (60.0–79.1)	74.9 (61.9–84.3)	0.0012
Age ≥ 65 years	478 (69.4)	231 (67.7)	247 (71.0)	0.357
Male gender, n (%)	393 (55.6)	181 (53.1)	202 (58.0)	0.190
Comorbidities, n (%)				
Obesity	149 (21.6)	68 (19.9)	92 (23.3)	0.288
CKD	138 (20.0)	73 (21.4)	65 (18.7)	0.371
Immune depression	151 (21.9)	113 (33.1)	38 (10.9)	<0.001
Transplant recipients	30 (4.3)	24 (7.0)	6 (1.7)	0.001
Diabetes	155 (22.5)	75 (22.0)	80 (23.0)	0.755
Chronic liver disease	47 (6.8)	28 (8.2)	19 (5.5)	0.152
COPD	114 (16.5)	68 (19.9)	46 (13.2)	0.018
Previous CVA	185 (26.8)	134 (39.3)	51 (14.7)	<0.001
Oncological disease	143 (20.8)	93 (27.3)	50 (14.4)	<0.001
Heamatological cancer	66 (9.6)	44 (12.9)	22 (6.3)	0.003
Heamatological cancer with chemotherapy treatment	45 (6.5)	30 (8.8)	15 (4.3)	0.017
Cardiovascular disease	203 (29.5)	76 (22.3)	127 (36.5)	<0.001
Vaccine, n (%)	564 (81.8)	291 (85.3)	273 (78.4)	0.019
Time between last vaccine dose and SARS-CoV-2 positivity, median (IQR)	155 (96–213)	173 (105–223.5)	149 (85–199)	0.001
Time between last vaccine dose and of SARS-CoV-2 positivity ≤ 155 days, n (%)	282/564 (50.0)	132/291 (45.4)	150/273 (54.9)	0.023
Symptoms, n (%)	553 (80.3)	341 (100)	212 (60.9)	<0.001
Fever, n (%)	324 (47.0)	198 (58.1)	126 (36.2)	<0.001
Cough, n (%)	276 (40.1)	192 (56.3)	84 (24.1)	<0.001
Ageusia, n (%)	13 (1.9)	11 (3.2)	2 (0.6)	0.011
Sore throat, n (%)	145 (21.0)	121 (35.5)	24 (6.9)	<0.001
Asthenia, n (%)	233 (33.8)	150 (44.0)	83 (23.8)	<0.001
Myalgia, n (%)	102 (14.8)	74 (21.7)	28 (8.0)	<0.001
GI symptoms, n (%)	74 (10.7)	35 (10.3)	39 (11.2)	0.689
Dyspnea, n (%)	123 (17.8)	37 (10.8)	86 (24.7)	<0.001
Days between symptoms onset and SARS-CoV-2 diagnosis	1 (0–2)	1 (0–2)	1.5 (0–3)	<0.001
Monlupiravir	61 (8.85)	61 (17.9)	0	-
Nirmatrelvir/ritonavir	9 (1.3)	9 (2.6)	0	-
Remdesivir	26 (3.8)	26 (7.6)	0	-
Disease progression, n (%)	161 (23.4)	17 (5.0)	144 (41.4)	<0.001
Death, n (%)	81 (11.8)	18 (5.3)	63 (18.1)	<0.001
Death in people with disease progression, n (%)	65 (9.4)	9 (2.6)	56 (16.1)	<0.001

IQR: interquartile range; CKD: chronic kidney disease; COPD: chronic obstructive pulmonary disease; CVA: cerebrovascular accident; GI: gastrointestinal.

**Table 2:** Logistic regression analysis to assess the relationship between demographics, clinical characteristics, and need to start oxygen therapy.

	Univariate Analysis		Multivariate Analysis	
Variables	OR (95%CI)	*p*-Value	OR (CI95%)	*p*-Value
Age (10 years)	1.26 (1.11–1.43)	<0.001	1.23 (1.04–1.45)	0.015
Obesity	1.53 (1.02–2.29)	0.038	1.19 (0.69–2.06)	0.528
Immunodeficit	0.55 (0.34–0.88)	0.012	1.59 (0.82–3.01)	0.168
Previous CVA	0.47 (0.30–0.74)	0.001	1.12 (0.62–2.03)	0.701
Oncological disease	0.47 (0.28–0.78)	0.003	0.95 (0.49–1.86)	0.891
Cardiovascular disease	2.20 (1.52–3.19)	<0.001	1.69 (1.01–2.80)	0.044
Vaccine, n (%)	0.22 (0.14–0.33)	<0.001	0.21 (0.12–0.37)	<0.001
Symptoms, n (%)	3.15 (1.76–5.66)	<0.001		
Fever, n (%)	1.76 (1.23–2.51)	0.002	3.88 (2.35–6.38)	<0.001
Dyspnea, n (%)	9.15 (5.94–14.07)	<0.001	7.24 (4.17–12.58)	<0.001
Sotrovimab	0.07 (0.4–0.13)	<0.001	0.05 (0.02–0.11)	<0.001
Sotrovimab + Antiviral	0.22 (0.10–0.49)	<0.001	1.47 (0.51–4.21)	0.476

OR: odd ratio; CI: confidence interval; CVA: cerebrovascular accident.

**Table 3:** Logistic regression analysis to assess the relationship between demographics, clinical characteristics, and death due to COVID-19.

	Univariate Analysis		Multivariate Analysis	
Variables	OR (95%CI)	*p*-Value	OR (95%CI)	*p*-Value
Age (10 years)	1.48 (1.20–1.83)	<0.001	1.36 (1.09–1.69)	0.006
Haematological cancer with chemotherapy treatment	1.86 (0.79–4.35)	0.152	4.07 (1.45–11.4)	0.008
Cardiovascular disease	2.75 (1.64–4.62)	<0.001	1.76 (0.96–3.22)	0.065
Vaccine, n (%)	0.33 (0.19–0.57)	<0.001	0.37 (0.20–0.68)	0.002
Dyspnea, n (%)	5.27 (3.09–9.00)	<0.001	3.63 (2.02–6.50)	<0.001
Sotrovimab	0.14 (0.07–0.29)	<0.001	0.16 (0.06–0.42)	<0.001
Sotrovimab + Antiviral	0.38 (0.13–1.07)	0.066	1.79 (0.45–7.16)	0.412

## Data Availability

The data that support the findings of this study are available from the corresponding author, upon reasonable request.

## References

[1] Chan J.F.W., Kok K.H., Zhu Z., Chu H., To K.K.W., Yuan S., Yuen K.Y. (2020). Genomic Characterization of the 2019 Novel Human-Pathogenic Coronavirus Isolated from a Patient with Atypical Pneumonia after Visiting Wuhan. Emerg. Microbes Infect..

[2] Cevik M., Tate M., Lloyd O., Maraolo A.E., Schafers J., Ho A. (2020). SARS-CoV-2, SARS-CoV, and MERS-CoV Viral Load Dynamics, Duration of Viral Shedding, and Infectiousness: A Systematic Review and Meta-Analysis. Lancet Microbe.

[3] Pormohammad A., Ghorbani S., Khatami A., Farzi R., Baradaran B., Turner D.L., Turner R.J., Bahr N.C., Idrovo J. (2020). Comparison of Confirmed COVID-19 with SARS and MERS Cases—Clinical Characteristics, Laboratory Findings, Radiographic Signs and Outcomes: A Systematic Review and Meta-analysis. Rev. Med. Virol..

[4] Lamers M.M., Haagmans B.L. (2022). SARS-CoV-2 Pathogenesis. Nat. Rev. Microbiol..

[5] Mittal A., Manjunath K., Ranjan R.K., Kaushik S., Kumar S., Verma V. (2020). COVID-19 Pandemic: Insights into Structure, Function, and HACE2 Receptor Recognition by SARS-CoV-2. PLoS Pathog..

[6] De Vito A., Fiore V., Princic E., Geremia N., Panu Napodano C.M., Muredda A.A., Maida I., Madeddu G., Babudieri S. (2021). Predictors of Infection, Symptoms Development, and Mortality in People with SARS-CoV-2 Living in Retirement Nursing Homes. PLoS ONE.

[7] Vaira L.A., Deiana G., Fois A.G., Pirina P., Madeddu G., De Vito A., Babudieri S., Petrocelli M., Serra A., Bussu F. (2020). Objective Evaluation of Anosmia and Ageusia in COVID-19 Patients: A Single-center Experience on 72 Cases. Head Neck.

[8] Geremia N., De Vito A., Gunnella S., Fiore V., Princic E., Panu Napodano C., Madeddu G., Babudieri S. (2020). A Case of Vasculitis-Like Skin Eruption Associated With COVID-19. Infect. Dis. Clin. Pract..

[9] Grant M.C., Geoghegan L., Arbyn M., Mohammed Z., McGuinness L., Clarke E.L., Wade R.G. (2020). The Prevalence of Symptoms in 24,410 Adults Infected by the Novel Coronavirus (SARS-CoV-2; COVID-19): A Systematic Review and Meta-Analysis of 148 Studies from 9 Countries. PLoS ONE.

[10] Vaira L.A., De Vito A., Deiana G., Pes C., Giovanditto F., Fiore V., Lechien J.R., Saussez S., Policicchio D., Boccaletti R. (2021). Systemic Inflammatory Markers and Psychophysical Olfactory Scores in Coronavirus Disease 2019 Patients: Is There Any Correlation?. J. Laryngol. Otol..

[11] Vaira L.A., De Vito A., Deiana G., Pes C., Giovanditto F., Fiore V., Lechien J.R., Le Bon S.D., Saussez S., Madeddu G. (2022). Correlations between IL-6 Serum Level and Olfactory Dysfunction Severity in COVID-19 Patients: A Preliminary Study. Eur. Arch. Otorhinolaryngol.

[12] Drake T.M., Riad A.M., Fairfield C.J., Egan C., Knight S.R., Pius R., Hardwick H.E., Norman L., Shaw C.A., Mclean K.A. (2021). Characterisation of In-Hospital Complications Associated with COVID-19 Using the ISARIC WHO Clinical Characterisation Protocol UK: A Prospective, Multicentre Cohort Study. Lancet.

[13] De Vito A., Geremia N., Fiore V., Princic E., Babudieri S., Madeddu G. (2020). Clinical Features, Laboratory Findings and Predictors of Death in Hospitalized Patients with COVID-19 in Sardinia, Italy. Eur. Rev. Med. Pharmacol. Sci..

[14] Zinellu A., De Vito A., Scano V., Paliogiannis P., Fiore V., Madeddu G., Maida I., Zinellu E., Mangoni A.A., Arru L.B. (2021). The PaO_2_/FiO_2_ Ratio on Admission Is Independently Associated with Prolonged Hospitalization in COVID-19 Patients. J. Infect. Dev. Ctries..

[15] Duong D. (2021). Alpha, Beta, Delta, Gamma: What’s Important to Know about SARS-CoV-2 Variants of Concern?. CMAJ Can. Med. Assoc. J..

[16] Scovino A.M., Dahab E.C., Vieira G.F., Freire-de-Lima L., Freire-de-Lima C.G., Morrot A. (2022). SARS-CoV-2’s Variants of Concern: A Brief Characterization. Front. Immunol..

[17] Nealon J., Cowling B.J. (2022). Omicron Severity: Milder but Not Mild. Lancet.

[18] Sigal A., Milo R., Jassat W. (2022). Estimating Disease Severity of Omicron and Delta SARS-CoV-2 Infections. Nat. Rev. Immunol..

[19] Mohsin M., Mahmud S. (2022). Omicron SARS-CoV-2 Variant of Concern: A Review on Its Transmissibility, Immune Evasion, Reinfection, and Severity. Medicine.

[20] Mader A.L., Tydykov L., Glück V., Bertok M., Weidlich T., Gottwald C., Stefl A., Vogel M., Plentz A., Köstler J. (2022). Omicron’s Binding to Sotrovimab, Casirivimab, Imdevimab, CR3022, and Sera from Previously Infected or Vaccinated Individuals. iScience.

[21] De Vito A., Colpani A., Bitti A., Zauli B., Meloni M.C., Fois M., Denti L., Bacciu S., Marcia C., Maida I. (2022). Safety and Efficacy of Molnupiravir in SARS-CoV-2 Infected Patients: A Real-Life Experience. J. Med. Virol..

[22] De Vito A., Colpani A., Saderi L., Puci M., Zauli B., Fiore V., Fois M., Meloni M.C., Bitti A., Di Castri C. (2023). Impact of Early SARS-CoV-2 Antiviral Therapy on Disease Progression. Viruses.

[23] Mazzitelli M., Mengato D., Sasset L., Ferrari A., Gardin S., Scaglione V., Bonadiman N., Calandrino L., Cavinato S., Trivellato S. (2023). Molnupiravir and Nirmatrelvir/Ritonavir: Tolerability, Safety, and Adherence in a Retrospective Cohort Study. Viruses.

[24] Olliaro P., Torreele E., Vaillant M. (2021). COVID-19 Vaccine Efficacy and Effectiveness—The Elephant (Not) in the Room. Lancet Microbe.

[25] De Vito A., Colpani A., Trunfio M., Fiore V., Moi G., Fois M., Leoni N., Ruiu S., Babudieri S., Calcagno A. (2023). Living with HIV and Getting Vaccinated: A Narrative Review. Vaccines.

[26] VanBlargan L.A., Errico J.M., Halfmann P.J., Zost S.J., Crowe J.E., Purcell L.A., Kawaoka Y., Corti D., Fremont D.H., Diamond M.S. (2022). An Infectious SARS-CoV-2 B.1.1.529 Omicron Virus Escapes Neutralization by Therapeutic Monoclonal Antibodies. Nat. Med..

[27] Touret F., Baronti C., Bouzidi H.S., de Lamballerie X. (2022). In Vitro Evaluation of Therapeutic Antibodies against a SARS-CoV-2 Omicron B.1.1.529 Isolate. Sci. Rep..

[28] Takashita E., Yamayoshi S., Halfmann P., Wilson N., Ries H., Richardson A., Bobholz M., Vuyk W., Maddox R., Baker D.A. (2022). In Vitro Efficacy of Antiviral Agents against Omicron Subvariant BA.4.6. N. Engl. J. Med..

[29] Araf Y., Akter F., Tang Y.d., Fatemi R., Parvez M.S.A., Zheng C., Hossain M.G. (2022). Omicron Variant of SARS-CoV-2: Genomics, Transmissibility, and Responses to Current COVID-19 Vaccines. J. Med. Virol..

[30] Chenchula S., Karunakaran P., Sharma S., Chavan M. (2022). Current Evidence on Efficacy of COVID-19 Booster Dose Vaccination against the Omicron Variant: A Systematic Review. J. Med. Virol..

[31] Post L.A., Lorenzo-Redondo R. (2022). Omicron: Fewer Adverse Outcomes Come with New Dangers. Lancet.

[32] Iuliano A.D., Brunkard J.M., Boehmer T.K., Peterson E., Adjei S., Binder A.M., Cobb S., Graff P., Hidalgo P., Panaggio M.J. (2022). Trends in Disease Severity and Health Care Utilization During the Early Omicron Variant Period Compared with Previous SARS-CoV-2 High Transmission Periods-United States, December 2020–January 2022. MMWR Morb. Mortal. Wkly. Rep..

[33] Ministero Della Salute—Istituto Superiore di Sanità (2022). Aggiornamento Casi COVID-19—Dati AggregatiQuotidianiRegioni/PPAA.

[34] Istituto Superiore di Sanità Comunicato Stampa N°08/2022-Covid-19, Flash Survey Iss: Il 17 Gennaio Il 95,8% Dei Campioni Positivi a Omicron. https://www.iss.it/web/guest/cov19-cosa-fa-iss-varianti/-/asset_publisher/yJS4xO2fauqM/content/comunicato%C2%A0stampa-n%C2%B008-2022-covid-19-flash-survey-iss-il-17-gennaio-il-95-8-dei-campioni-positivi-a-omicron?_com_liferay_asset_publisher_web_portlet_AssetPublisherPortlet_INSTANCE_yJS4xO2fauqM_assetEntryId=6608164&_com_liferay_asset_publisher_web_portlet_AssetPublisherPortlet_INSTANCE_yJS4xO2fauqM_redirect=https%3A%2F%2Fwww.iss.it%2Fweb%2Fguest%2Fcov19-cosa-fa-iss-varianti%3Fp_p_id%3Dcom_liferay_asset_publisher_web_portlet_AssetPublisherPortlet_INSTANCE_yJS4xO2fauqM%26p_p_lifecycle%3D0%26p_p_state%3Dnormal%26p_p_mode%3Dview%26_com_liferay_asset_publisher_web_portlet_AssetPublisherPortlet_INSTANCE_yJS4xO2fauqM_assetEntryId%3D6608164%26_com_liferay_asset_publisher_web_portlet_AssetPublisherPortlet_INSTANCE_yJS4xO2fauqM_cur%3D0%26p_r_p_resetCur%3Dfalse.

[35] Poliseno M., Drago E.P., Poli M.A., Altamura M., Bruno S.R., Calamo A., Giannelli A., Infante G., Mazzola M., Moschetta D. (2023). Clinical Characteristics, Outcomes, and Risk Factors of Patients Hospitalized for COVID-19 across the Latest Pandemic Waves: Has Something Changed?. BioMed.

[36] Hoffmann M., Krüger N., Schulz S., Cossmann A., Rocha C., Kempf A., Nehlmeier I., Graichen L., Moldenhauer A.S., Winkler M.S. (2022). The Omicron Variant Is Highly Resistant against Antibody-Mediated Neutralization: Implications for Control of the COVID-19 Pandemic. Cell.

[37] Gupta A., Gonzalez-Rojas Y., Juarez E., Crespo Casal M., Moya J., Falci D.R., Sarkis E., Solis J., Zheng H., Scott N. (2021). Early Treatment for Covid-19 with SARS-CoV-2 Neutralizing Antibody Sotrovimab. N. Engl. J. Med..

[38] Piccicacco N., Zeitler K., Ing A., Montero J., Faughn J., Silbert S., Kim K. (2022). Real-World Effectiveness of Early Remdesivir and Sotrovimab in the Highest-Risk COVID-19 Outpatients during the Omicron Surge. J. Antimicrob. Chemother..

[39] Cheng M.M., Reyes C., Satram S., Birch H., Gibbons D.C., Drysdale M., Bell C.F., Suyundikov A., Ding X., Maher M.C. (2023). Real-World Effectiveness of Sotrovimab for the Early Treatment of COVID-19 During SARS-CoV-2 Delta and Omicron Waves in the USA. Infect. Dis. Ther..

[40] Chavarot N., Melenotte C., Amrouche L., Rouzaud C., Sberro-Soussan R., Pavie J., Martinez F., Pouvaret A., Leruez-Ville M., Cantin D. (2022). Early Treatment with Sotrovimab Monoclonal Antibody in Kidney Transplant Recipients with Omicron Infection. Kidney Int..

[41] Radcliffe C., Palacios C.F., Azar M.M., Cohen E., Malinis M. (2022). Real-World Experience with Available, Outpatient COVID-19 Therapies in Solid Organ Transplant Recipients during the Omicron Surge. Am. J. Transplant..

[42] Pinchera B., Buonomo A.R., Scotto R., Carrano R., Salemi F., Galluccio F., Guarino M., Viceconte G., Schiano Moriello N., Giaccone A. (2022). Sotrovimab in Solid Organ Transplant Patients With Early, Mild/Moderate SARS-CoV-2 Infection: A Single-Center Experience. Transplantation.

[43] Evans A., Qi C., Adebayo J.O., Underwood J., Coulson J., Bailey R., Lyons R., Edwards A., Cooper A., John G. (2023). Real-World Effectiveness of Molnupiravir, Nirmatrelvir-Ritonavir, and Sotrovimab on Preventing Hospital Admission among Higher-Risk Patients with COVID-19 in Wales: A Retrospective Cohort Study. J. Infect..

[44] Woo M.S., Brehm T.T., Fischer M., Heyer A., Wichmann D., Jordan S., Nörz D., Lütgehetmann M., Addo M.M., Lohse A.W. (2023). Sotrovimab in Hospitalized Patients with SARS-CoV-2 Omicron Variant Infection: A Propensity Score-Matched Retrospective Cohort Study. Microbiol. Spectr..

[45] Aggarwal N.R., Beaty L.E., Bennett T.D., Carlson N.E., Davis C.B., Kwan B.M., Mayer D.A., Ong T.C., Russell S., Steele J. (2022). Real-World Evidence of the Neutralizing Monoclonal Antibody Sotrovimab for Preventing Hospitalization and Mortality in COVID-19 Outpatients. J. Infect. Dis..

[46] De Vito A., Colpani A., Madeddu G. (2023). Shedding New Light on COVID-19 Therapeutics during the Omicron Era: A Deeper Dive into Real-World Data. Lancet Reg. Health Eur..

